# Closing the Control Loop with Time-Variant Embedded Soft Sensors and Recurrent Neural Networks

**DOI:** 10.1089/soro.2021.0012

**Published:** 2022-12-12

**Authors:** Thomas George Thuruthel, Paul Gardner, Fumiya Iida

**Affiliations:** The Bio-Inspired Robotics Lab, Department of Engineering, University of Cambridge, Cambridge, United Kingdom.

**Keywords:** soft sensors, closed-loop control, machine learning, force control, recurrent neural networks

## Abstract

Embedded soft sensors can significantly impact the design and control of soft-bodied robots. Although there have been considerable advances in technology behind these novel sensing materials, their application in real-world tasks, especially in closed-loop control tasks, has been severely limited. This is mainly because of the challenge involved with modeling a nonlinear time-variant sensor embedded in a complex soft-bodied system. This article presents a learning-based approach for closed-loop force control with embedded soft sensors and recurrent neural networks (RNNs). We present learning protocols for training a class of RNNs called long short-term memory (LSTM) that allows us to develop accurate and robust state estimation models of these complex dynamical systems within a short period of time. Using this model, we develop a simple feedback force controller for a soft anthropomorphic finger even with significant drift and hysteresis in our feedback signal. Simulation and experimental studies are conducted to analyze the capabilities and generalizability of the control architecture. Experimentally, we are able to develop a closed-loop controller with a control frequency of 25 Hz and an average accuracy of 0.17 N. Our results indicate that current soft sensing technologies can already be used in real-world applications with the aid of machine learning techniques and an appropriate training methodology.

## Introduction

Soft robots and their derived technologies provide a new paradigm for developing intelligent, safe, and robust robotic systems.^1,2^ Among them, soft robots with embedded soft sensors are important for proprioception, exteroception, and for developing feedback controllers.^3^ Although the area of soft robotic sensors has seen significant developments in the recent years, the usage of these technologies in real-world applications has been limited.^4^ The key reason for this is the difficulty in modeling these nonlinear time-variant dynamic systems, which is further complicated by the uncertainties introduced by the fabrication process.^5^ For feedback control applications, fast, accurate, and robust models of these sensors are required, which are addressed in this work.

Typically, feedback control of soft robotic systems is performed using external sensory systems.^6^ This removes/reduces the need for modeling the sensory system itself and focuses only on the dynamics of the actuator-body system. Recently, both model-based^7^ and model-free^8,9^ approaches have shown promise in solving the closed-loop dynamic control problem. However, these systems are limited in the type of control task they can perform due to their limited sensing capabilities. Internal body states like stress are not measurable using the current external sensing technologies like optical tracking. Moreover, they are adversely affected by occlusion problems, especially in tasks involving contact. Hence, embedded sensing technologies are important for tasks involving force control and dexterous manipulation.

Embedded sensing using soft robotic sensors has immense potential to advance tactile sensing abilities in robotic systems.^4,5^ Due to their high compliance and omnidirectional response they can be easily combined to measure contact, deformation, pressure, and stress. These sensors are made with materials that provide an electrical stimulus in response to a mechanical input. Resistive and piezoresistive sensors change their electrical conductivity in response to mechanical stimulus. They can be developed with conductive liquids,^10^ elastomeric composites with conductive fillers,^11,12^ conductive yarn,^13^ etc. Other modes of sensing technologies like capacitive^14,15^ and optical sensing^16,17^ have been widely studied too. This work uses the commonly used resistive-type strain sensors.

Once the sensors are embedded in the system, the next problem is to estimate the states of the robot from the sensor signals. The complexity of the state estimation problem is not only dependent on the electromechanical properties of the sensor material itself but also on the structure of the sensor and its dynamic interaction with the embedding matrix. For linear time-invariant sensors simple analytical models are sufficient, especially when characterizing the sensor by itself.

Learning based approaches are typically used for modeling these sensors once they are embedded in a surrounding matrix irrespective of their sensing mechanism. They have been used for human body tracking,^18–22^ proprioception,^23–26^ grasp prediction,^27^ tactile sensing,^28–30^ etc. Nonetheless, the most convincing example of applicability and robustness of these architectures comes through a closed-loop control task. In this study, we need accurate and fast sensing methods that are robust over a wide range of motion bandwidth and interactions.

To close the control loop with these embedded soft sensors, it is necessary to have a model that maps from the control inputs and the controllable state of the robot on top of the state-estimation model. The state-of-the-art in closed-loop control of soft robots relies on collocated sensor-actuator structures to simplify this problem.^31^ Collocated architectures rely on aligned sensor-actuator pairs.^32,33^ This leads to a monotonic relationship between the sensor signal and the control variable, which allows us to control the system with simple proportional–integral–derivative (PID) controller.^34–36^ However, such designs are highly restrictive on the design space of the soft robotic system and difficult to implement for passive and underactuated systems. In addition, if there are time variant nonlinearities present in the system, such control architectures would not work.

This article presents a closed-loop control architecture for a passive underactuated anthropomorphic arm with nonlinear time variant embedded soft sensors (See Supplementary Video S1). Due to the complexity of the design, the arrangement of the sensors, and the temporal nonlinearities in the sensor, we develop a learning based control architecture that provides robust and accurate state estimation. With the learned model, we create a simple feedback controller for closed-loop force control at 25 Hz. Our work is the first demonstration of a closed-loop controller for a general soft robotic system with no assumptions on the location and linearity of the embedded soft sensors.

## Materials and Methods

### Experimental setup

**FIG. 1.**
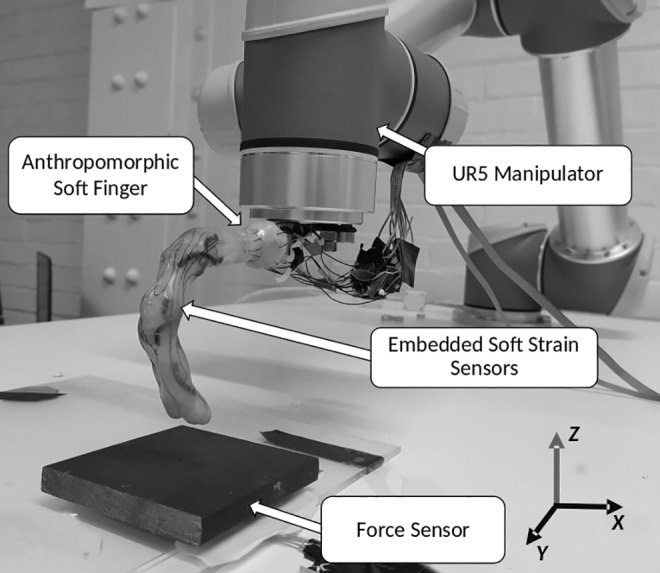
Experimental setup.

The experiments are conducted on a passive anthropomorphic finger with rigid skeleton, flexible ligaments, and a soft skin. More details on the fabrication of the finger can be found in the author's previous work.^37^ The skeleton of the finger is three-dimensional printed and attached with compliant joints. The skeleton is then spin coated with a layer of Ecoflex-10. The strain sensor strands are then placed in U-shapes on the skin with varying lengths. We use a resistive soft strain sensor called Conductive Thermoplastic Elastomer (CTPE) for the state feedback.^38^ They are a composite of a thermoplastic elastic matrix and carbon black powder made under high pressure and temperature. The passive finger is mounted on a UR5 industrial manipulator, which is constrained to move only in the *Z*-direction (Fig. 1).

A force sensitive resistor (FSR) is fixed in the *XY* plane for obtaining ground truth force data for training the soft sensors. The finger comes in contact with the FSR through a rigid plate, which is placed on the FSR. The raw signals from the soft sensors and the FSR are read using a 16-bit NI USB-6212 data acquisition system after going through a voltage divider. The data from the analog channels are read through USB by the MATLAB programming environment, and the UR5 robot is controlled through a TCP/IP connection. Internally, the UR5 robot is controlled by an end-effector trajectory following command called *servoj*.^39^ The *servoj* command is designed to execute smooth trajectories in the joint space by having a look ahead horizon. For our case, as we have a reactive controller, this look ahead horizon is restricted to a short period of two times the control frequency. A blocking time of 0.039 s is manually selected to make sure that the arm never comes to a complete rest while not lagging the commanded trajectory.

**FIG. 2.**
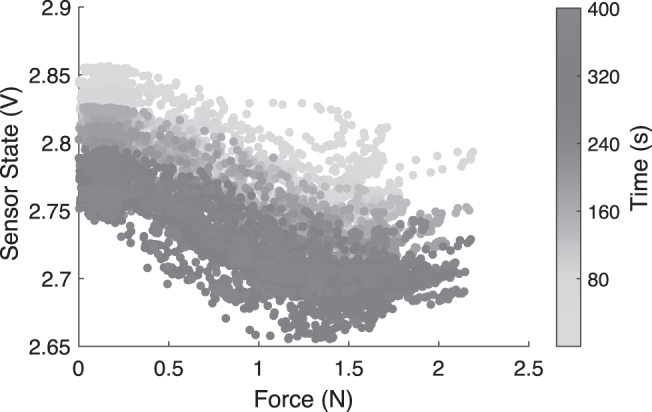
Behavior of a single embedded soft sensor to external force overtime.

The response of one of the embedded soft sensors to random motions in the allowed direction is shown in Figure 2, as a scatter plot. There are two key points to be observed here. First, as the system has a noncollocated architecture, it is not necessary to have a monotonic relationship between the sensor and the control variable, which in our case is the force in the *Z*-direction. Second, the sensor has significant temporal nonlinearities like drift and hysteresis. This makes the problem of estimating the actual force from the sensor state difficult. To address the first issue, we are using multiple sensors for estimation of the system state. For handling temporal nonlinearities, we use recurrent neural networks (RNNs) that utilize information from past data points for prediction.

### Sensor modeling

For modeling the relationship between sensor responses and the applied tip force by the finger we use long short-term memory (LSTM) RNNs.^40^ LSTMs are a type of RNNs that have been developed to deal with the vanishing gradient problem typically found in training traditional RNNs.^41^

Even though LSTM networks can be used to learn the temporal relation in the sensor data, there is still a problem in the initialization of the network. Consider the case of drift in the sensor data. Even if the sensors exhibit a predictable drift pattern, once the robotic device is turned off, the current drift state is lost. Hence, a LSTM network trained for a specific initial condition will perform poorly upon repeated experimentation. One way to solve this problem is to wait for a long period between each trial. However, this is limiting in the applicability of the device, and it is not necessary that all the sensors return to their initial state after the resting period.

To solve this problem, we use a stateful LSTM network for the prediction problem along with a batch sampling process that trains the network on various initial conditions.^42^ A stateful LSTM updates their internal state with every data in the input sequence. The training is obtained as 20 batches of sequence data that are obtained at varying sensor states. This is done by random motion of the finger in the *Z* direction for around 400 s for each batch. Successive batches are obtained by repeating the process after a wait time of 300 s. No data are obtained during the wait period. The whole data collection hence requires only 4 h. The sensor data from all the six sensors and the ground truth sensor are then resampled to 25 Hz for training, providing around 400000 data points.

**FIG. 3.**
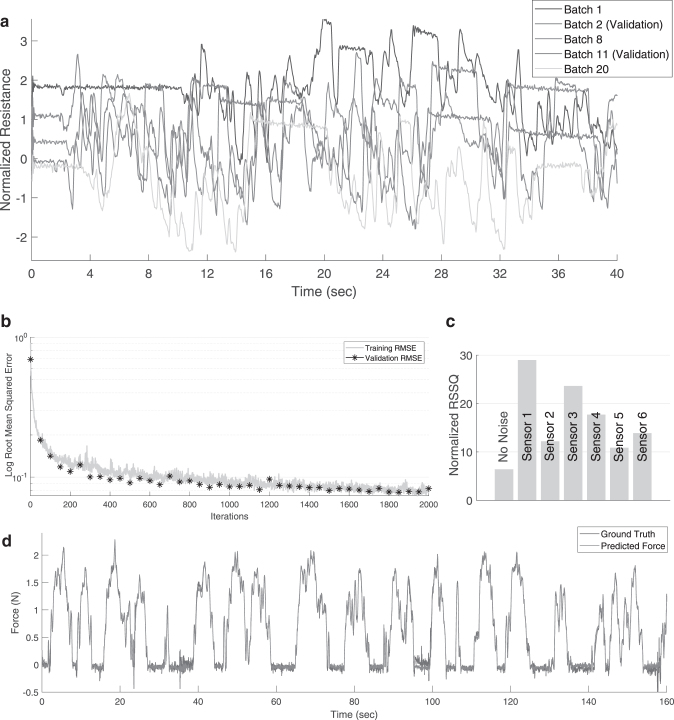
Sampling and learning data. **(a)** Raw sensor data for the batches obtained for training the LSTM network. Note that the initial value of the sensor varies even though the ground truth force value is the same. **(b)** LSTM training progress. **(c)** Role of the redundant sensor configuration on the prediction performance. **(d)** LSTM predictions on the validation set after training. LSTM, long short-term memory.

The whole data are then normalized by the mean and standard deviation of the combined batch data (The same mean and standard deviation values throughout the remaining tests). Two of the batches are used for validation during training and the rest for training. An example of the raw sensor data for one of the soft sensors is shown in Figure 3a. Note that the initial value of the sensor varies even though the ground truth force value is the same (The finger starts from no-contact configuration). The validation batches are taken near beginning and end of sampling to ensure that the model is not overfitting (Fig. 3a).

We use a single LSTM layer with 50 hidden units for learning the mapping between the 6 soft sensor data and the actual *Z*-axis force. A dropout layer is not used due to the relatively small size of the LSTM network with respect to the number of training data. A mini-batch size of 5 is used for updating the weights of the network after each iteration. The MATLAB toolbox for deep learning is used for creating and training the LSTM network. The Adam (adaptive moment estimation) optimizer is used for updating the weights of the network. The root mean squared error (RMSE) for the training and validation data is shown in Figure 3b. As the validation set is obtained independently from the training data, the reduction in validation RMSE with training RMSE is a strong indicator that there is no overfitting of the data.

The performance of the LSTM network after training can be seen through the predictions on the validation set, as shown in Figure 3d. The learned model is not only accurate and robust to the nonlinearities present in the sensors but also robust to the noise present in our ground truth data. The role of the redundant sensor data can be inferred by selectively adding noise to the sensor data on the validation set. The effect of adding a zero mean normally distributed noise with 0.02 standard deviation to each sensor data is shown in Figure 3c. Although some of the sensors are more important for prediction, all the sensors have some contribution to the final estimation.

### Control architecture

**FIG. 4.**
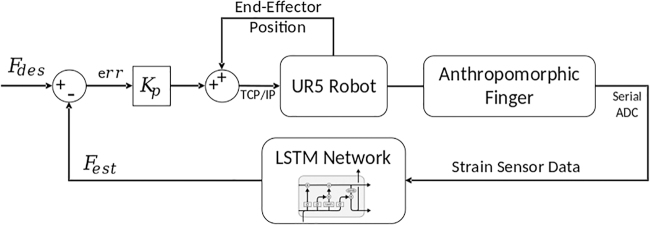
Control architecture for closed-loop force control with the LSTM network.

Although the relationship between the sensor signals and applied force is highly nonlinear and time variant, the mapping between the applied force and displacement of the base manipulator can be assumed to be monotonic. This allows to close the loop with a proportional feedback over the estimated and desired force values (Fig. 4). With a model of the relationship between displacement and applied force values, more accurate tracking can be obtained. The controller is run at 25 Hz, the same frequency as the learned model. The proportional gain is tuned manually. Due to the noise in the data, a derivative component is not added alongside the proportional component for the experimental studies. We perform simulation studies to investigate the advantages of a Proportional Derivative controller over a Proportional controller.

At the start of each test, the states of LSTM network are reset to its initial value. Note that due to our learning strategy, the LSTM network can be reset at any point, and we can expect the model predictions to be accurate after the initial few data points are fed to the system. At every control cycle the raw sensor from the six sensors are measured, resampled to the desired control frequency by linear interpolation, normalized, and fed to the LSTM network.

The LSTM network provides the estimate of applied tip force and updates its internal states. Hence, every next prediction can be performed with only the recent sensor data. However, extra care must be taken to avoid providing noisy data to the network to ensure that the network states do not diverge to inaccurate values. As the training of the network is done such that the network is not dependent on the initial sensor state, the LSTM network can be reset to ensure accurate predictions.

## Simulation Study

**FIG. 5.**
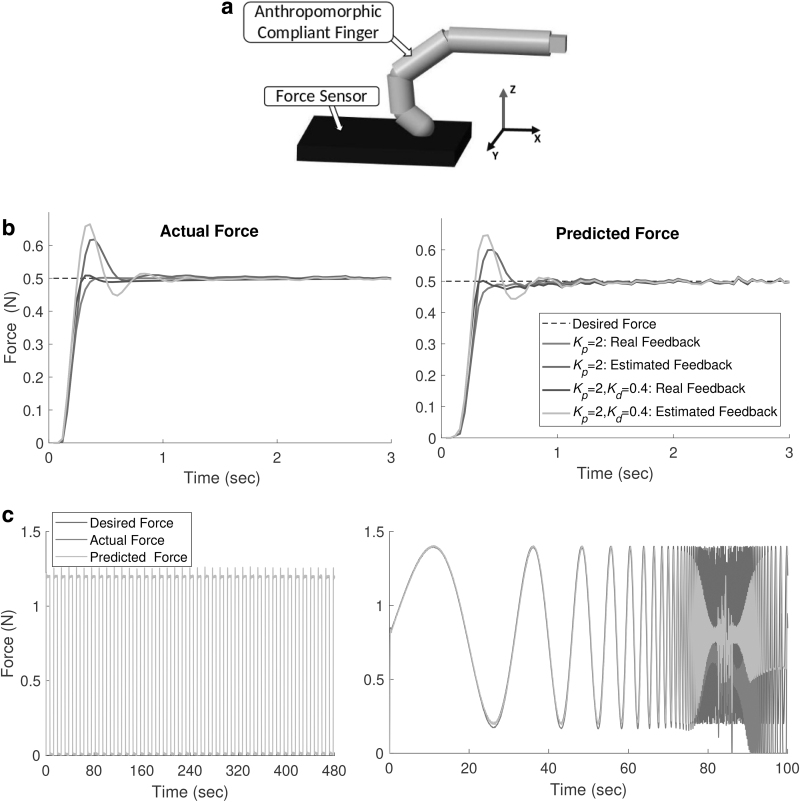
Closed-loop force control with the stimulated soft finger. **(a)** Simulated anthropomorphic finger. **(b)** Performance of the proportional feedback controller versus a proportional derivative controller. **(c)** Long term performance of the proposed control architecture and scenarios in which the learned model starts to deviate from the ground truth.

To test the generalizability of the approach and the limits of the controller, we perform control studies on a simulated anthropomorphic finger developed in *MATLAB Simscape* (Fig. 5a). Unlike the real setup, the simulated finger has fixed degrees of freedom with three compliant revolute joints. The sensors are simulated as a function that transforms all the three joint angle information into a single value with added nonlinear drift and noise. The drift is modeled using an ideal memristor with a nonlinear dopant drift approach. Eight such sensors are simulated with random noise and parameters for the memristor. The finger is constrained to move only in the *Z* direction, with a limit on the maximum acceleration based on real UR5 arm. The training protocol is kept the same for comparison purposes. The controller is also run at 25 Hz.

Figure 5b shows the effect of adding a derivative component to the feedback controller for both the learned state-estimation model and the ideal state feedback. As expected, with real feedback from the ground truth sensor, the feedback controller performs the best with little overshoot and faster convergence. Adding the derivative component improves the overall performance. Due to the added delay with the state-estimation model, the feedback control has a much higher overshoot for the same control parameters. Adding the derivative component does not improve the performance because of the added delay and noise in the sensor. Adding an integral term is not necessary here because of the low steady state error.

Figure 5c shows the long term performance of the controller and scenarios when the state-estimation model starts to deviate from the ground truth. For fixed-period force tracking experiments we do not see any performance degradation over time even after using the controller for periods longer than the training batch size. The prediction errors however deteriorate when the frequency of the tracked force profile increases. This is most likely because the training data, even though it is random, do not contain high frequency components. This is unavoidable as high frequency motions can cause damage to the system and has to be avoided in any random exploration process.

To show the generalizability of the approach we also develop a learned model to estimate the three joint angles of the finger along with the applied force at the tip. For a passive system, the relationship between joint angles and applied forces is a one-to-one mapping and can be easily estimated simultaneously. As the sensors are based on strain sensing, the state predictions would be affected by change in the dynamic properties of the finger.

**FIG. 6.**
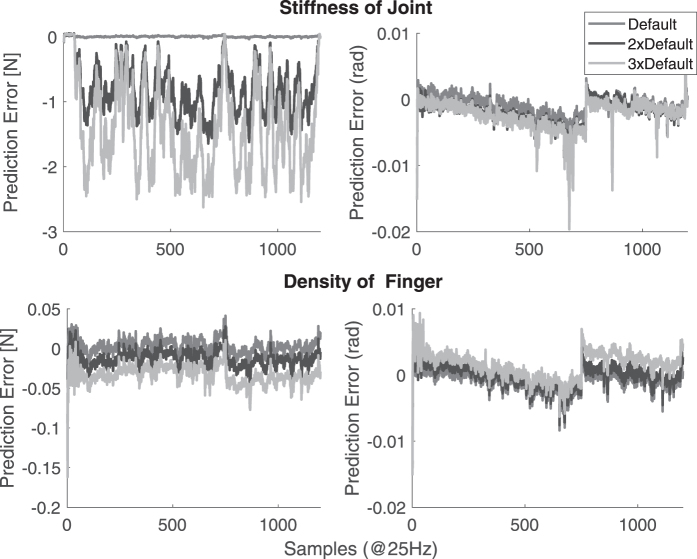
Change in prediction accuracy when the dynamic properties of the finger change without retraining.

Figure 6 shows how the force and joint angle predictions get affected with a change in stiffness and density of the joint and finger, respectively. As the joint angles are directly related to the strain values observed, change in stiffness does not affect the joint angle predictions. The external force estimation would however be affected by a change in joint stiffness. Other dynamic properties like change in damping and mass do not affect both the state estimation models significantly. So proper care must be taken in the applicability of the strain sensors for estimating external forces, especially in variable stiffness mechanisms, unlike kinematic state estimation.

## Experimental Results

Three sets of experimental tests are performed to validate the proposed control architecture. The first test investigates the effect of the proportional gain on the tracking performance. The second study measures the steady-state error of the force controller, and the third one examines the frequency response of the controller. All the results are displayed in force units after a simple calibration of the ground truth sensor with known weights and zero offsetting of the ground truth sensor at the start of each trial.

### Proportional gain tuning

**FIG. 7.**
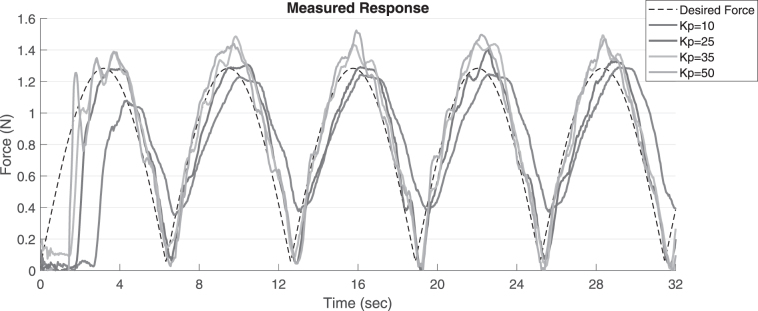
Tuning the proportional gain in the feedback controller.

The effect of the proportional gain on the tracking of a dynamic force profile is shown in Figure 7. All the data are filtered with a moving filter of 0.4 s window for clarity. As expected, higher gains lead to oscillations and increased instability, whereas lower gains lead to less accurate tracking. Hence, for all the dynamic experiments a proportional gain value of 25 was used, and for the steady state experiments, a proportional gain of 10 was used. Note that the overall stability of the controller is dependent on the accuracy of the state estimator, parameters of the PID controller, delays in the system, and the desired force profile.

**FIG. 8.**
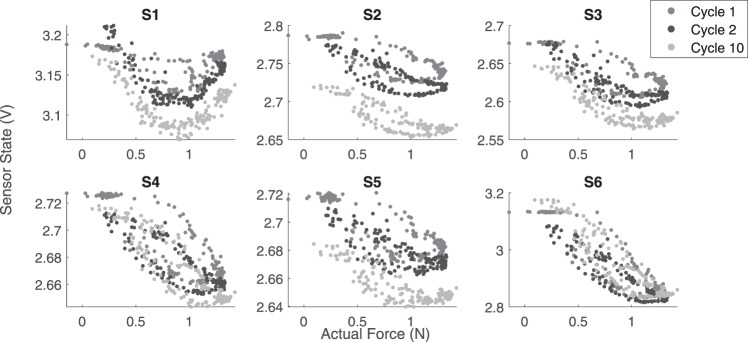
Sensor response to the periodic sine force profile shown in Figure 7.

Adding a derivative component can improve the stability and accuracy of the system, but it will be difficult due to the noise in the sensor data and the sensor delay as shown in the Simulation Study section. The sensor response to the periodic force profile over each cycle is shown in Figure 8 to illustrate the high temporal nonlinearity and noise in the system that must be compensated by the LSTM network. The average runtime for each component in the control architecture is shown in Table 1. The major delay introduced in the system is by the on-demand data acquisition system. This can be reduced significantly by parallel data acquisition and data synchronization; however, this is beyond the scope of this article. The next section presents the frequency response of the soft-bodied system.

**Table 1.** Runtime for Each Process in the Controller

Process	Average runtime (s)
Overall control loop	0.04 ± 0.004
Data acquisition	0.031 ± 0.004
LSTM prediction+ UR5 command time	0.009 ± 0.002

### Steady-state performance

**FIG. 9.**
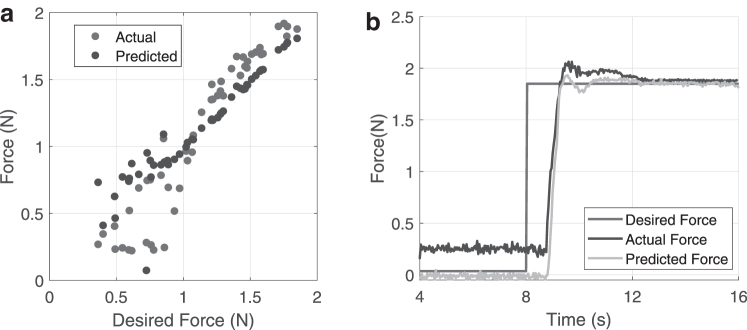
Steady-state response of the closed-loop force controller. **(a)** Controller performance to steady-state tracking of 50 random force inputs. **(b)** Example of a single steady-state force control experiment.

To measure the accuracy of the state estimator and the controller, we perform a constant force tracking at low control gain (*K_p_* = 10). Fifty random force values are selected and sent as commands to the closed-loop controller. The controller runs for 8 s (Fig. 9). The robot is reset to its zero position after every target, and the LSTM network is also reinitialized. There is no waiting time between each trial. The prediction and actual forces applied at the end of the control cycle are shown graphically in Figure 9a. As we are controlling the forces applied by the finger tip on the environment, there can be a lag from zero applied force to a positive force value, depending on how far the finger is from the external environment.

Errors caused by the inaccuracies in the LSTM prediction can be observed by the mismatch between the actual-predicted data points. The errors caused by the improper design of the PID controller and the UR5 controller can be observed by the deviations in the predicted data points from the unity-slope line. Note that the tracking of the predicted force with respect to the desired force is independent of the accuracy of the state estimator. The average error at the end of the control cycle for the 50 points was 0.17 ± 0.14 N. An example of the closed-loop tracing is shown in Figure 9b. Even though the ground truth sensor is zeroed at the beginning of the experiment, there can sometimes be shifts in the ground truth values due to the interfacing of the FSR. This can also affect the sample data for learning. We have ignored the errors incurred by this in our analysis. This can be easily rectified using better ground truth force sensors. Note that the response time of the controller is dependent on how far the finger is from contact with the environment.

### Frequency sweep

**FIG. 10.**
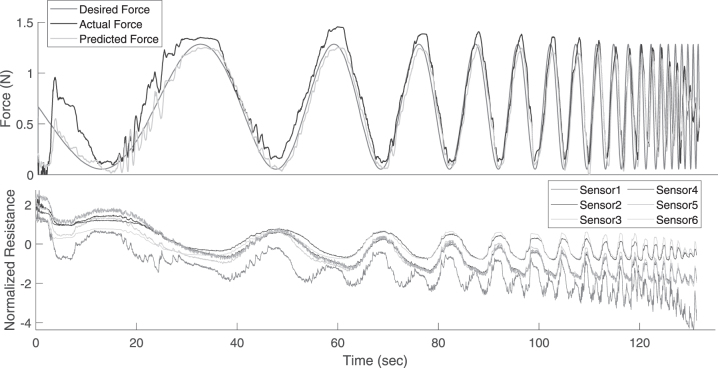
Time response of the close-loop force controller to a frequency sweep.

The frequency response of the closed-loop controller is affected by the dynamics of the sensors, the soft-bodied system, the PID controller, and the robotic manipulator. As any viscoelastic material will act like a low-pass filter, it would significantly reduce the zero motion bandwidth. However, there are several other advantages gained in terms of stability, energy storage, etc.^43^ The force tracking on a chirp signal is shown in Figure 10. All the data are filtered with a moving filter of 0.4 s window for clarity. The prediction accuracy of the LSTM network is not affected by the frequency of motion indicating that the learned model has generalized very well, even to high frequency motions.

Unlike the simulation studies, we restrict our motions to a certain frequency limit to avoid damages to the system. Some offset between the ground truth and predicted can be seen. This is probably because of the shifts in the ground truth values due to the interfacing of the FSR as mentioned in previous section. Using a well calibrated force sensor instead should reduce this error. The controller has a cutoff frequency of around 1 Hz. The corresponding sensor data from which the LSTM network predicts the true force values are shown in Figure 10. We can see that all the sensors drift by varying amounts and have varying sensitivity to the applied force. This is because the sensors are placed in random configurations and hence undergo different strain profiles.

## Conclusion

This article presents a learning based approach for closed-loop force control with embedded soft sensors on a general robotic system using the temporal modeling properties of RNNs. To the best of our knowledge, this work is the first step toward the development of a closed-loop controller for a noncollocated soft robotic system with embedded soft sensors. The key idea behind the process is the use of stateful LSTM networks and a batch sampling process that allows the learned network to be reinitialized at any point. With the learned LSTM network, a simple proportional feedback controller is designed to close the force control loop. The performance of the overall control architecture is then analyzed for both static and dynamic targets.

A straightforward extension of our work is to expand the controller for multiaxis force control. This would require additional sensing elements embedded in the system for similar accuracy and a multiaxis force sensor for the ground truth data. Another topic of future interest is the incorporation of other sensing modalities to improve the prediction of the LSTM network. This could be in the form of visual^44^ or base force sensor data.^45^ The current setup can be modified to solve high-level control tasks that require force information as a feedback signal. This can be done by adding a hierarchical control policy on top of the LSTM model or directly learning the control policy parameterized as a RNN.

## Data Availability Statement

Source code and data for this article can be found in: https://github.com/tomraven1/Closed-loop-sensors

## Supplementary Material

Supplemental data
